# Identifying Organisational Factors Related to Increased Risk of Depression in Usher Syndrome Patients: A Case Report

**DOI:** 10.1192/bjo.2024.671

**Published:** 2024-08-01

**Authors:** Fiona Kwan

**Affiliations:** University of Sheffield, Sheffield, United Kingdom

## Abstract

**Aims:**

Usher syndrome (USH) is the leading genetic aetiology of congenital hearing loss and progressive vision loss. It is linked with a high prevalence of mental health issues, including depression. Previous literature attribute this to communication barriers, constraints in mobility, and general feelings of dependency and uncertainty. However, there is little literature considering poor mental health in USH patients as a consequence of gaps in service provision on a national level.

**Methods:**

The present report is the case of a 54 year old woman, who was born with USH Type IIa, and was previously diagnosed with depression and retinitis pigmentosa. The patient was recruited via the RareBeacons charity through volunteer sampling. A semi-structured interview was conducted, with 3 main categories: the impact of diagnosis, interpersonal relationships, and challenges in day-to-day life. A common theme of self-isolation was found, largely due to inefficient communication between healthcare providers, including but not limited to years of waiting for hearing aid treatment exacerbating symptoms of social withdrawal. The patient also reported inadequacies in physician knowledge regarding USH and their general unwillingness to be educated further. Unprofessional physician attitudes and lack of sensitivity towards the patient's deafblindness over time led the patient to feel distrust towards the system, which further compromises care.

**Results:**

Areas of improvement on a systemic scale were identified, including increasing awareness of deafblindness in both the medical community and the general public through patient advocacy, as well as streamlining dedicated support pathways. The patient found formal support to be unhelpful, conversely emphasising the impact of informal support, namely web-based support group platforms. Support groups can provide a sense of community and belonging, alongside sharing valuable resources – often overlooked yet vital in USH, a rare condition with little official support. Subsequent research may include expansion of this case report to yield quantitative data, alongside investigating further factors increasing depression in USH patients (e.g. psychosocial, genetic and biological factors).

**Conclusion:**

This report concludes that the gaping inadequacies of the current medical system poses a significant psychological, emotional and social burden on USH patients.

